# A Case of Transfusion

**Published:** 1878-10

**Authors:** 


### EXCERPTA.

A CASE OF TRANSFUSION.—The patient was suffering from biliary colic, and during the passage of a calculus an intestinal vessel was ruptured. The treatment was the usual one adopted in biliary colic, with the addition of remedies directed to the suppression of the hemorrhage, which was controlled at the end of the second day—fully two quarts having been passed "per rectum," and a comparatively small quantity vomited. Two weeks later hemorrhage again occurred. More than a quart of clots, presenting perfect casts of the intestines, were passed at once. The next day another large quantity of blood and another perfect cast, measuring thirty-six inches. The patient was now fast sinking; extremities cold and bloodless. The doctor now (March 27th) determined upon the transfusion of blood. No pulse could be felt at the wrist, and the heart was beating 155. Veins would not fill when the arm was ligated; 4 oz. of defibrinated human blood was transfused, and at the conclusion of the operation the pulse beat 115; the face and fingers showed marked color, and the patient expressed himself as better. Convalescence has been slow and irregular, but without returning colic or hemorrhage, and is now considered perfect.—*N. Y. Medical and Surgical Brief.*

